# Supplementary material for: Developmental YAPdeltaC determines adult pathology in a model of spinocerebellar ataxia type 1
Source: Nat Commun. 2017 Nov 30;8:1864. doi: 10.1038/s41467-017-01790-z (PMC5709507; doi:10.1038/s41467-017-01790-z)
Supplement: Supplementary file 1 — Supplementary Information [file 41467_2017_1790_MOESM1_ESM.pdf]

Supplementary Figure 1

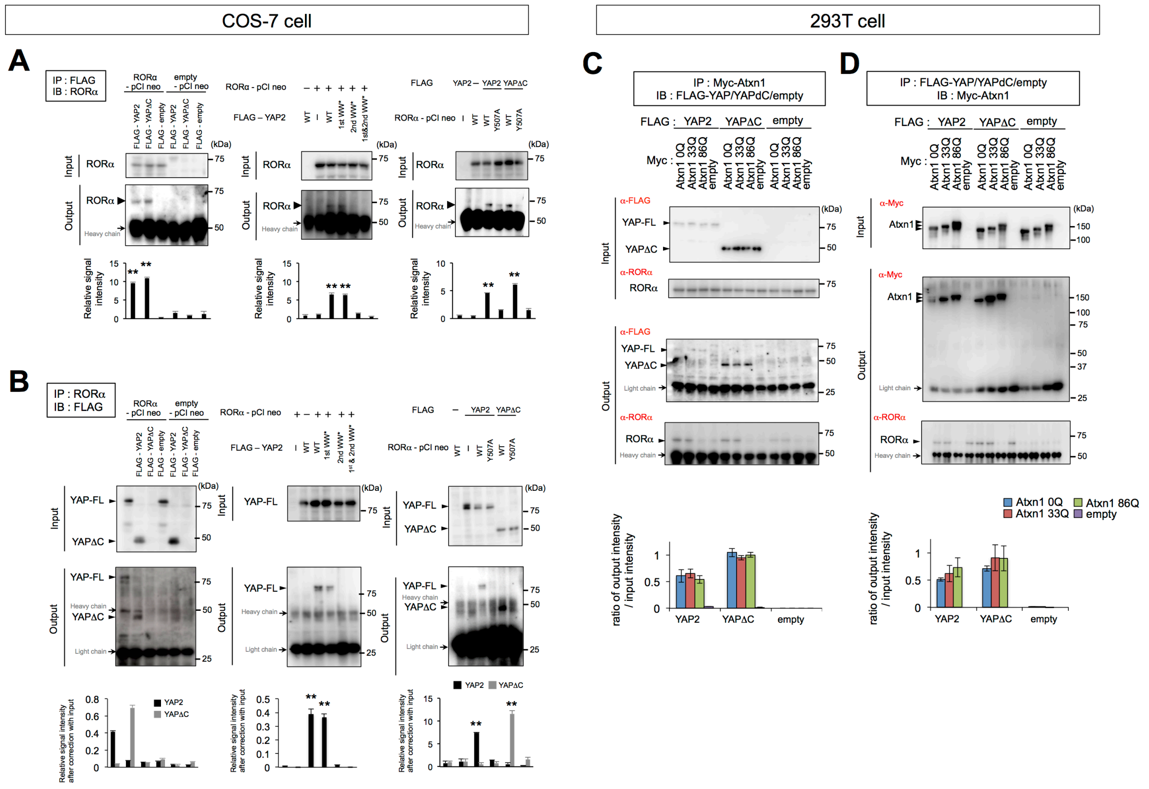

Supplementary Figure 1

Normal and mutant Atxn1 interact with YAP/YAPdeltaC

A, B) Immunoprecipitation analyses were performed in COS-7 cells, which hardly express RORα, transiently expressing RORα (no-tag) and FLAG-YAP (full-length YAP2) at 48 hours after transfection, to test the interaction of exogenous RORα with YAP or YAPdeltaC.

The lower graphs show quantitative analyses of the relative intensities of output bands, corrected based on the corresponding input band (N=3). Double asterisks: $p < 0.01$ in one-way ANOVA with post-hoc Tukey's HSD test.

C, D) Immunoprecipitation analyses were performed in 293T cells at 48 hours after transient transfection of FLAG-YAP2/YAPdeltaC and Myc-Atxn1-0Q/33Q/86Q. The results show that normal and mutant Atxn1 interacted with YAP2/YAPdeltaC with similar affinities. The interaction of YAPdeltaC with normal or mutant Atxn1 tended to be stronger than that of full-length YAP2.

Supplementary Figure 2

A

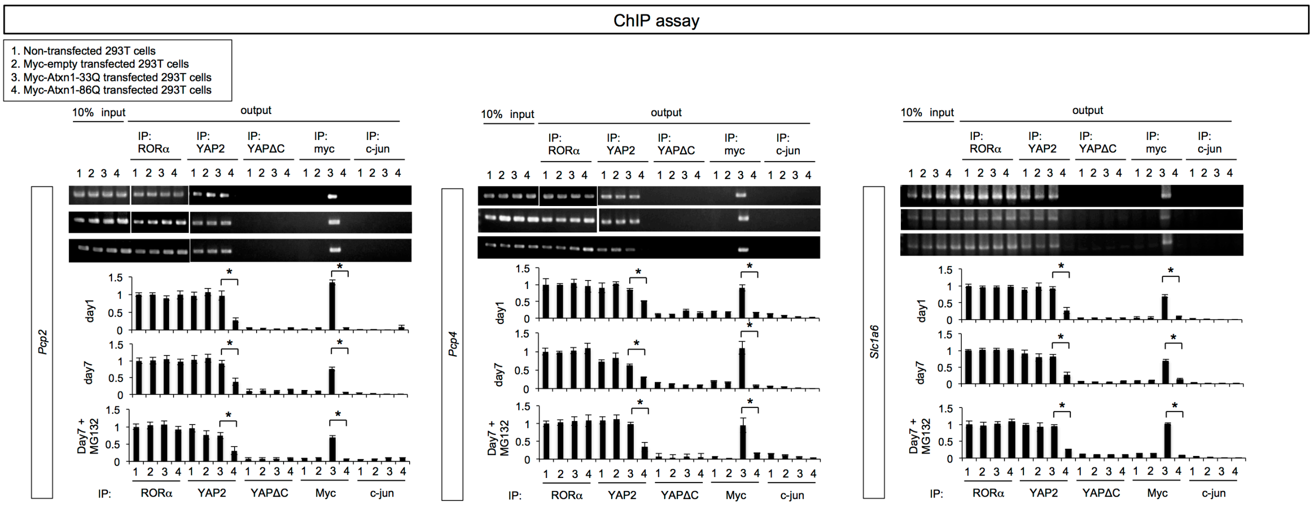

B

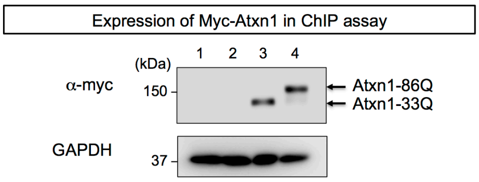

Supplementary Figure 2

Mutant Atxn1 depletes YAP and YAPdeltaC from the transcriptional complex on RORα-responsive elements

A) ChIP assays to examine the effect of Myc-Atxn1-33/86Q on RORα located on its cis-element in target genes (*Pcp2*, *Pcp4*, *Slc1a6*) and on the transcription complex containing Myc-Atxn1-33/86Q and RORα located on the cis-element. The amount of transcription complex on the cis-element was decreased by expression of Myc-Atxn1-86Q, whereas mutant Atxn1 did not change the amount of RORα attached to the cis-element. Precipitation with c-Jun was used as a negative control. Double asterisks indicate statistical significance ($p < 0.01$, N=5) in one-way ANOVA with post-hoc Tukey's HSD test.

B) Protein levels of Myc-Atxn1-33Q and Myc-Atxn1-86Q were tested by western blot with anti-Myc antibody using the samples shown in Supplementary Fig. 2A.

Supplementary Figure 3

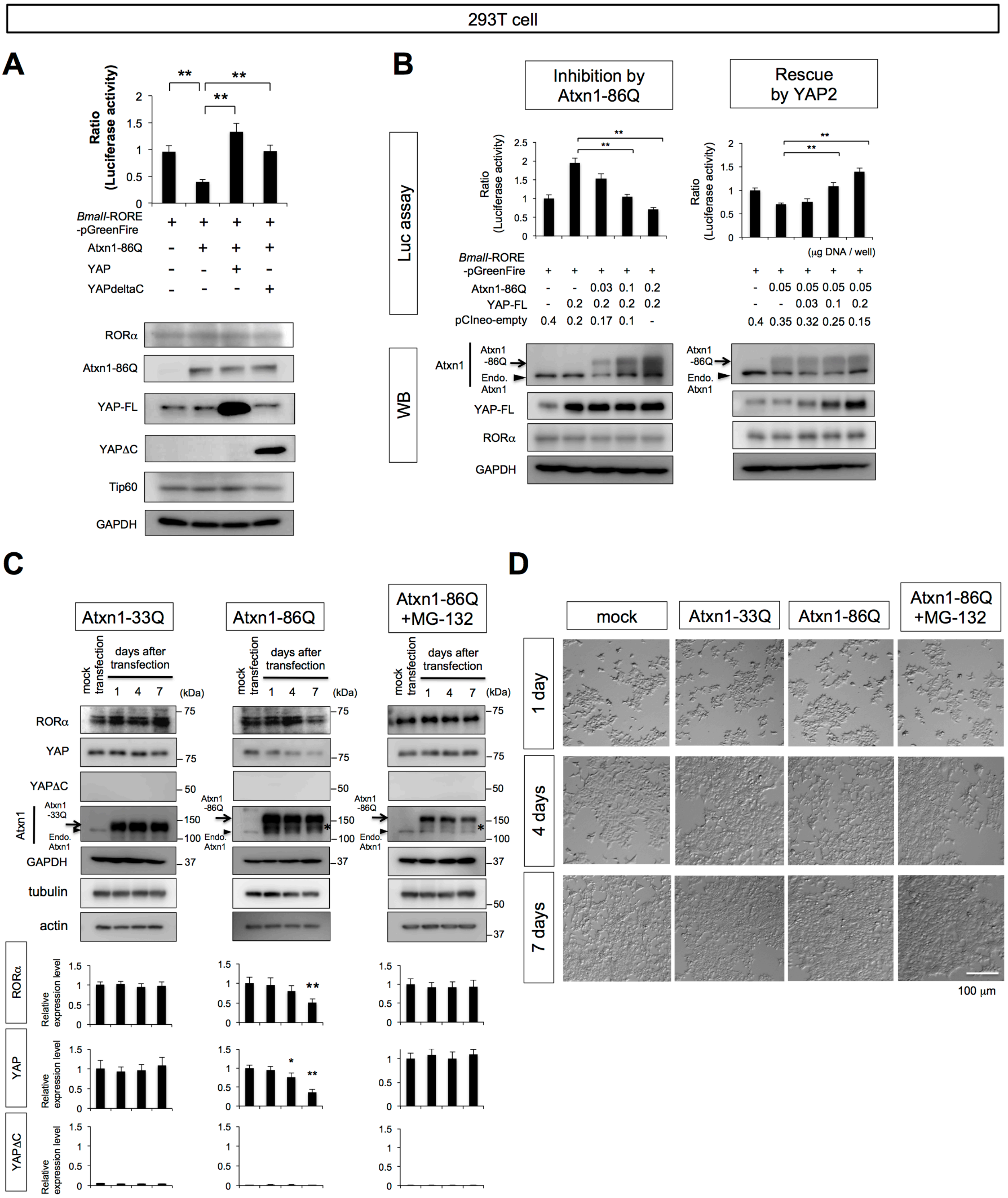

Supplementary Figure 3

YAP/YAPdeltaC restores RORα-target gene expression *in vitro*

A) In 293T cells, luciferase assays were performed at 1 day after transfection of reporter and effector plasmids (upper graph). Expression levels of RORα, Atxn1-86Q, YAP, YAPdeltaC, Tip60, and GAPDH at the time of luciferase assays are shown in the lower panels. Atxn1-86Q suppressed RORα-mediated transcription immediately, without decreasing the level of RORα or YAP. The

transcriptional suppression was rescued by co-expression of YAP or YAPdeltaC. Double asterisks indicate statistical significance ($p < 0.01$, N=7) in One-way ANOVA with post-hoc Tukey's HSD test.
B) Dose-dependent inhibitory effect of Atxn1-86Q (left panels), and rescue effect of YAP2 (right panels), on RORα-mediated transcription. Double asterisks indicate statistical significance ($p < 0.01$, N=5) in one-way ANOVA with post-hoc Tukey's HSD test.
C) Protein levels of RORα, YAP, YAPdeltaC, Atxn1, GAPDH, tubulin, and actin were continuously checked until 7 days after transfection. MG-132 treatment (0.3 μM) increased the level of Atxn1-86Q and decreased the intensity of a lower band (*) below Atxn1-86Q, which could represent a degradation intermediate. The results of quantitative analyses are shown. Double asterisks indicate statistical significance ($p < 0.01$, N=5) in Dunnett's test.
D) Phase-contrast images of 293T cells after transfection. Cell death was not markedly induced by mutant Atxn1 under the conditions used for the luciferase transcription assay.

Supplementary Figure 4

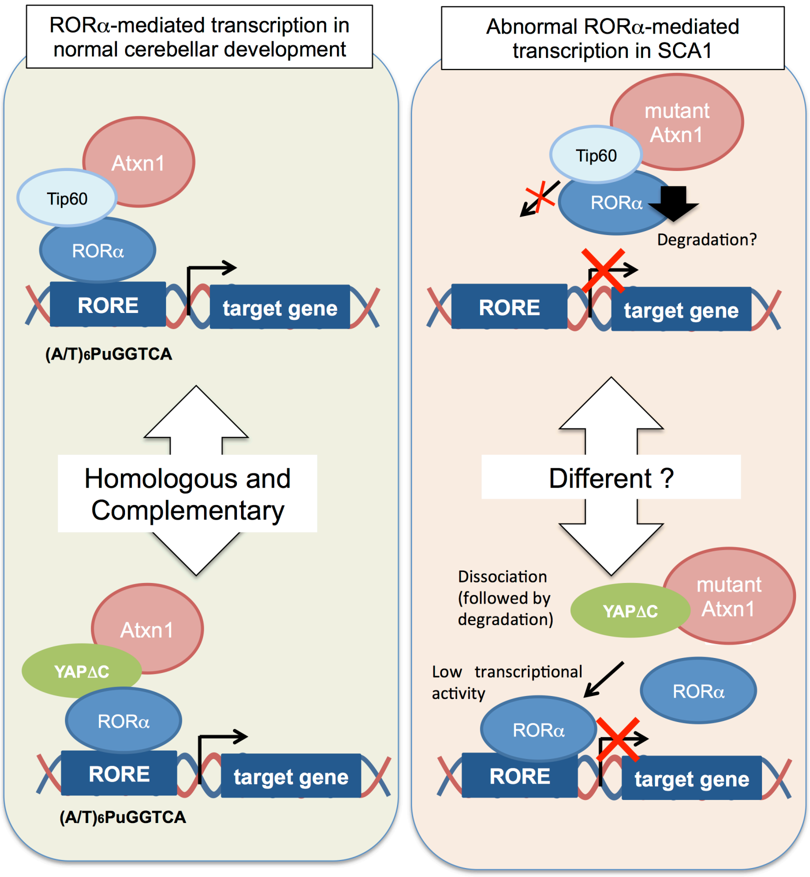

Supplementary Figure 4

Comparison of the roles of Tip60 and YAPdeltaC in RORα transcription under normal and SCA1 conditions

Model showing similarities and difference in the roles of Tip60 and YAPdeltaC in RORα transcription under normal and SCA1 conditions.

Supplementary Figure 5

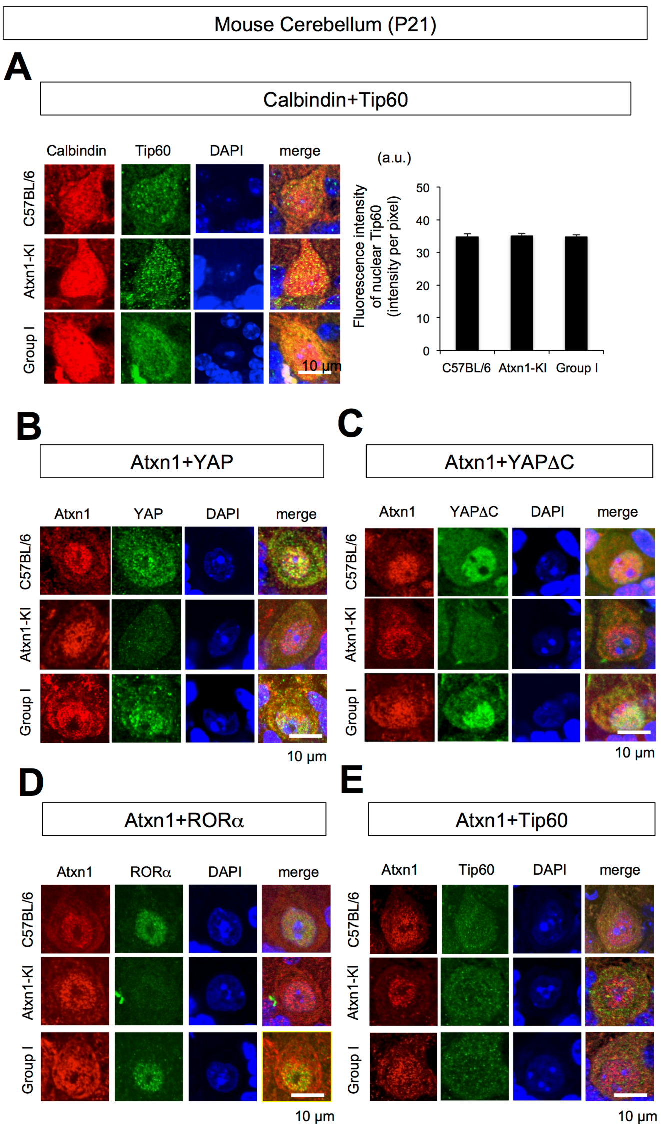

Supplementary Figure 5

Co-expression of YAP, YAPdeltaC, Tip60, RORα, and Atxn1 in Purkinje cells at P21

A) Calbindin-positive Purkinje cells were evaluated for Tip60 signal intensity in three groups of mice at 21 days of age (P21). Graphs at right show the results of quantitative analyses of Tip60 in the nuclei of Purkinje cells. Signal intensities were acquired from more than 100 Purkinje cells randomly selected in 30 slides from six mice in each group. One-way ANOVA with post-hoc Tukey's HSD test was used for statistical analysis. **$p < 0.01$.

B–E) Representative Purkinje cells co-stained with Atxn1 and YAP, YAPdeltaC, RORα or Tip60. The slides were co-stained with anti-Atxn1 antibody (11NQ, Millipore).

Supplementary Figure 6

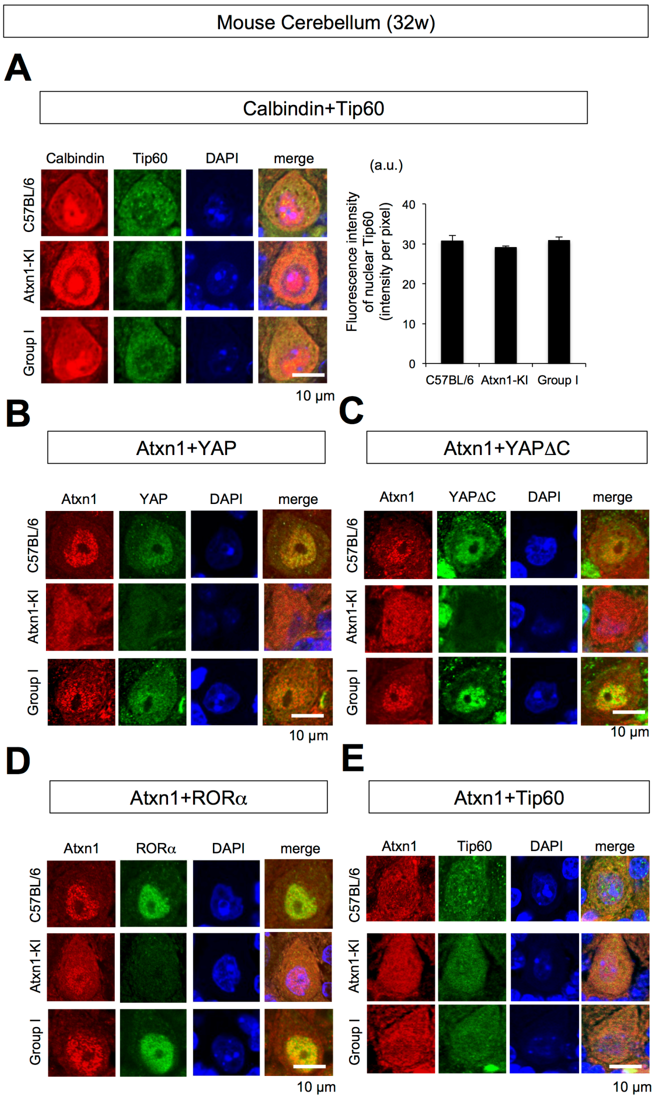

Supplementary Figure 6

Co-expression of YAP, YAPdeltaC, Tip60, RORα, and Atxn1 in Purkinje cells at 32 weeks

A) Calbindin-positive Purkinje cells were evaluated for Tip60 signal intensity in three groups of mice at 32 weeks of age (32w). Graphs at right show the results of quantitative analyses of Tip60 in the nuclei of Purkinje cells. Signal intensities were acquired from more than 100 Purkinje cells randomly selected in 30 slides from six mice in each group. One-way ANOVA with post-hoc Tukey's HSD test was used for statistical analysis. $**p < 0.01$.

B–E) Representative Purkinje cells co-stained with Atxn1 and YAP, YAPdeltaC, RORα or Tip60. The slides were co-stained with anti-Atxn1 antibody (11NQ, Millipore).

Supplementary Figure 7

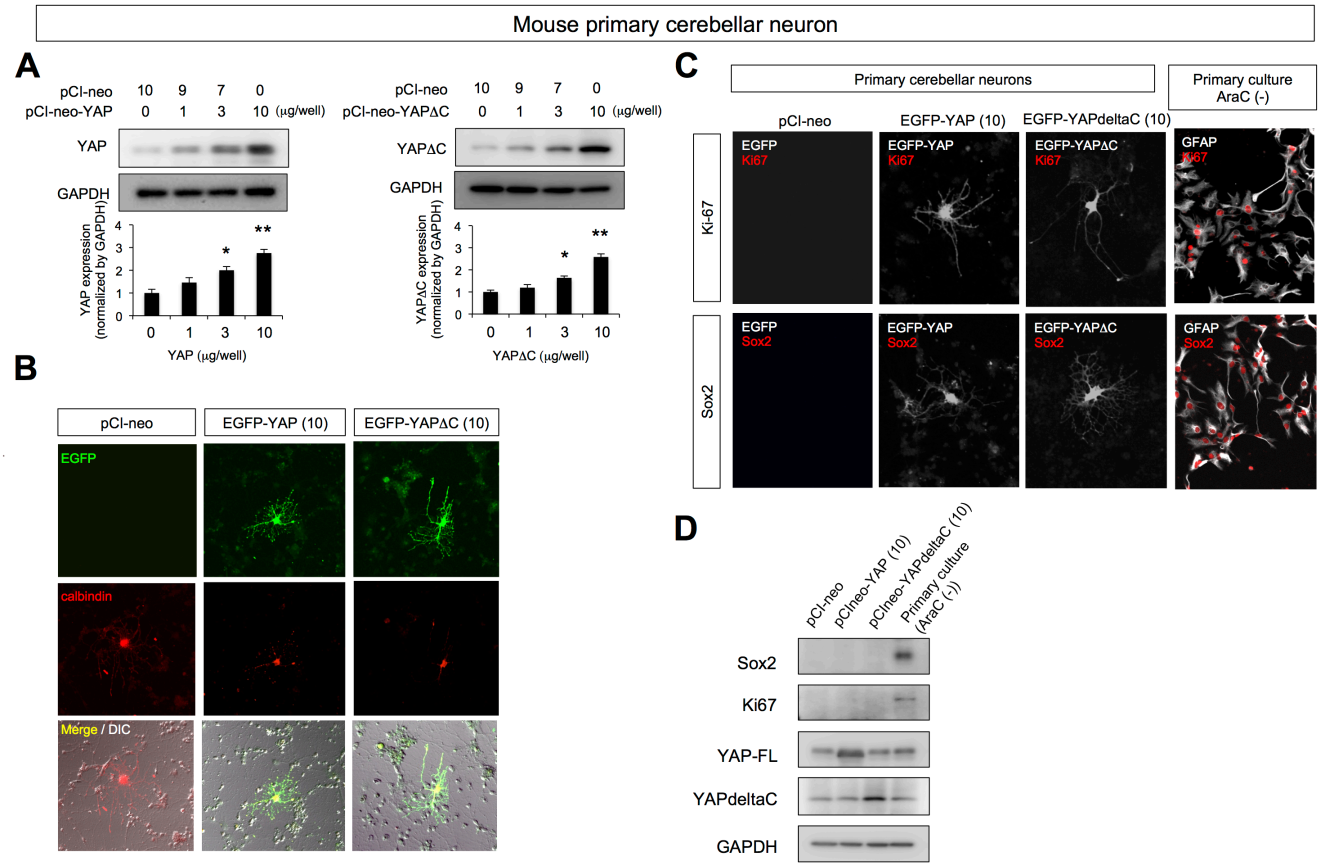

Supplementary Figure 7

YAP and YAPdeltaC do not induce tumorigenesis or de-differentiation of cerebellar neurons

A) Western blot analyses show different expression levels of YAP and YAPdeltaC in primary cerebellar neurons at 7 days after transfection.

B) No abnormal proliferation or morphological change was observed in Purkinje cells and other neurons after transfection of YAP and YAPdeltaC in primary cerebellar neurons until 14 days after transfection. DIC: differential interference contrast.

C) Immunocytochemistry of Ki67 or Sox2 did not detect proliferating or de-differentiating cells in YAP and YAPdeltaC in primary cerebellar neurons until 14 days after transfection.

D) Western blot analyses did not detect Ki67 or Sox2 in primary cerebellar neurons at 14 days after transfection of YAP and YAPdeltaC.

Supplementary Figure 8

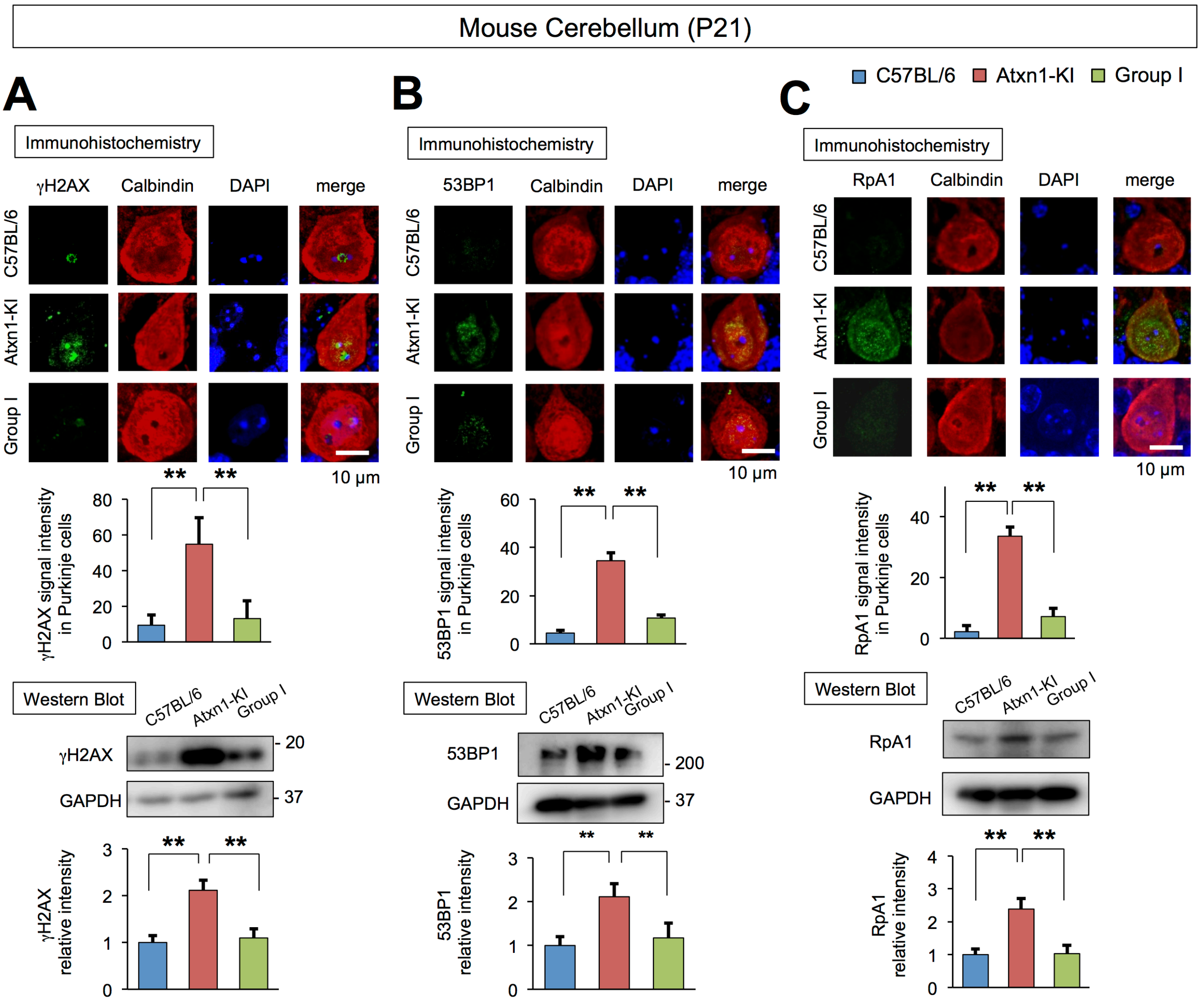

Supplementary Figure 8

YAPdeltaC recovers DNA damage in Atxn1-KI mice

Immunohistochemistry reveals that DNA damage markers γH2AX (A), 53BP1 (B) and RpA1 (C) were more abundant in Atxn1-KI mice at P21, but were restored to normal levels by YAPdeltaC expression in Group I mice. Signal intensities were quantitatively analyzed, and data are shown in the lower graphs. Signal intensities were acquired from more than 100 Purkinje cells randomly selected in 30 slides from six mice in each group. Asterisks and double asterisks indicate statistical significance ($p < 0.05$ and $p < 0.01$, respectively) in one-way ANOVA with post-hoc Tukey's HSD test. Western blot analysis revealed that band intensities of γH2AX (A), 53BP1 (B), and RpA1 (C) were elevated in Atxn1-KI mice at P21, but were decreased by YAPdeltaC expression in Group I mice. Band intensities, normalized against GAPDH, from four independent blots were analyzed, and the results are shown in the lower graphs.

Supplementary figure 9

Uncropped images

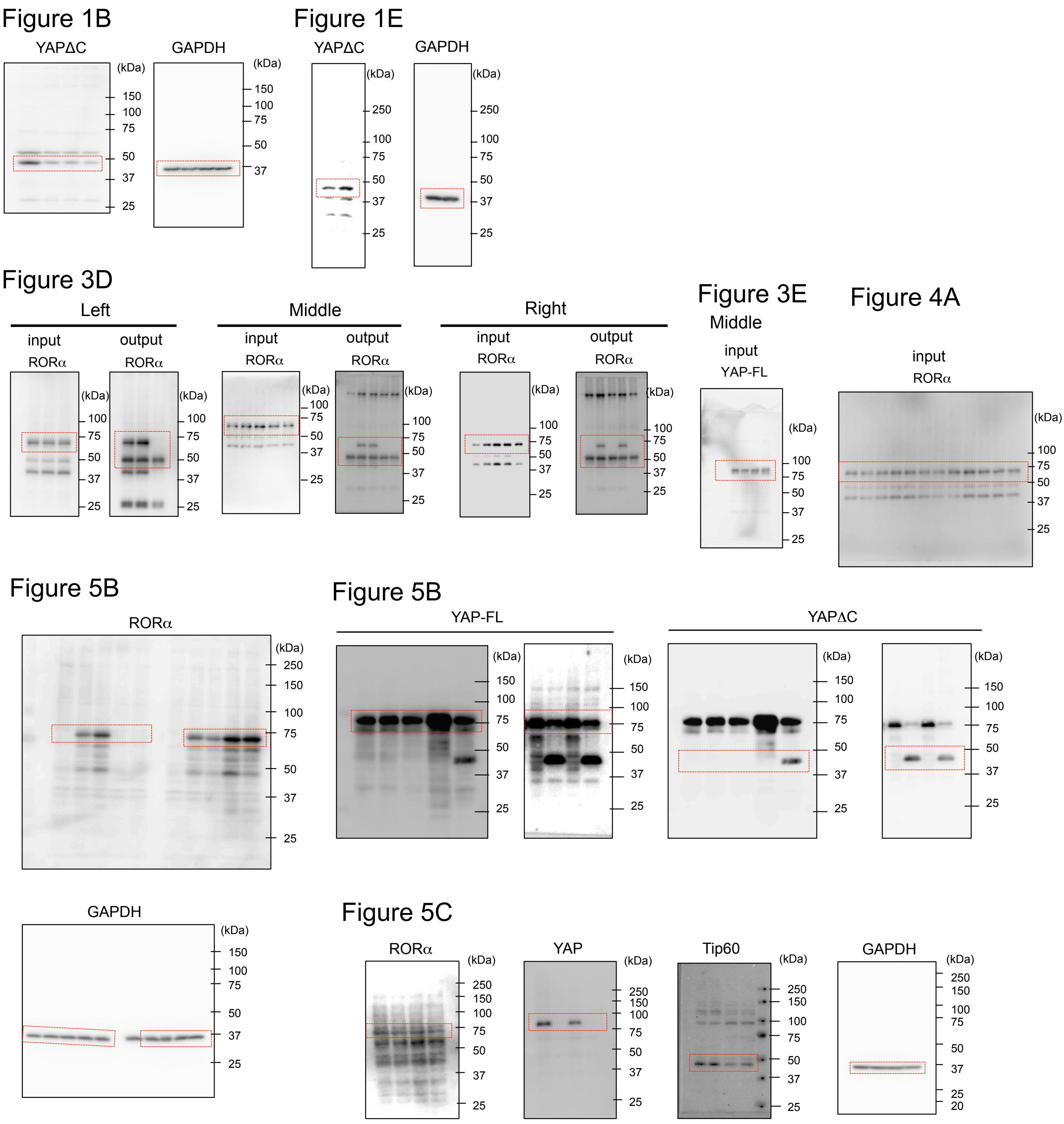

Figure 5D

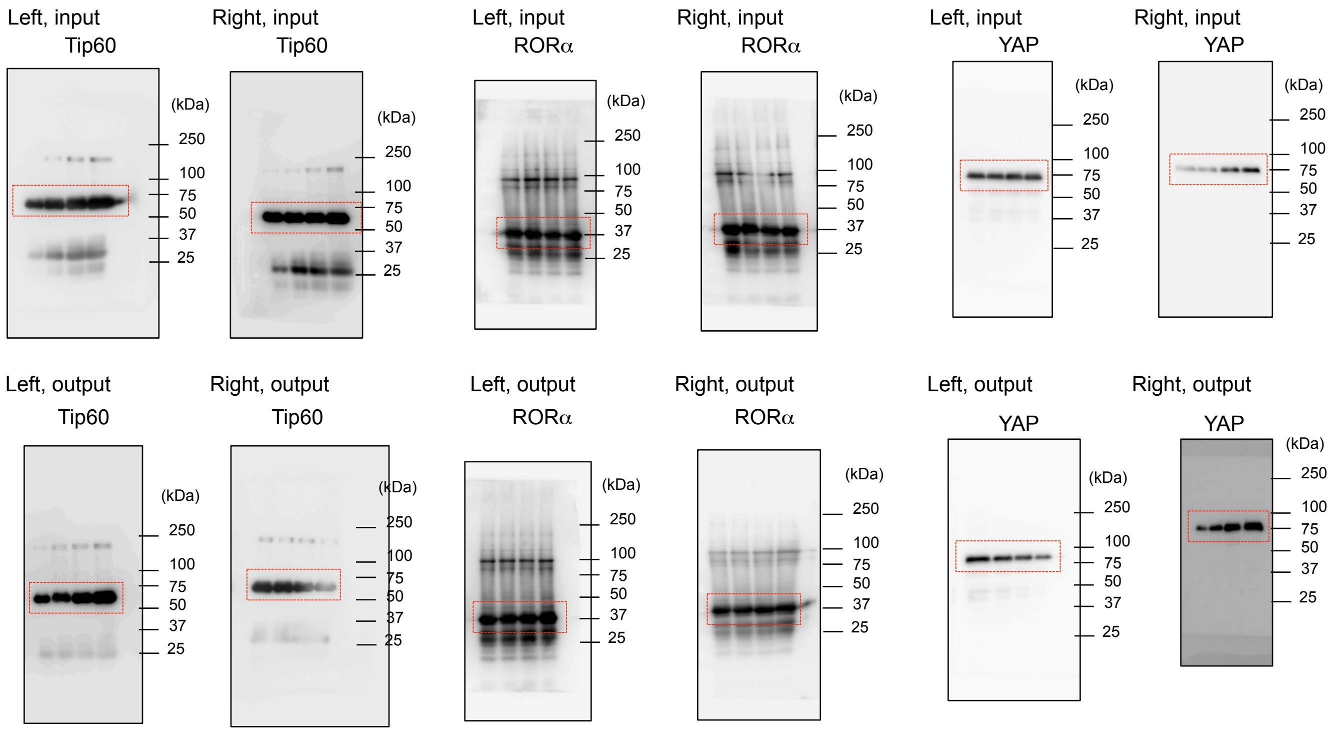

Figure 5E

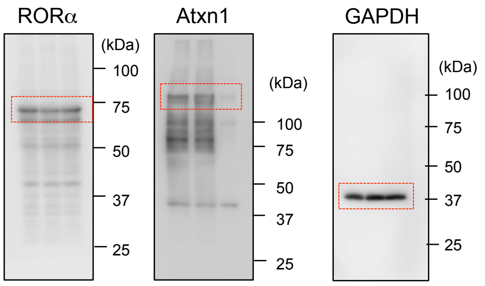

Figure 6A

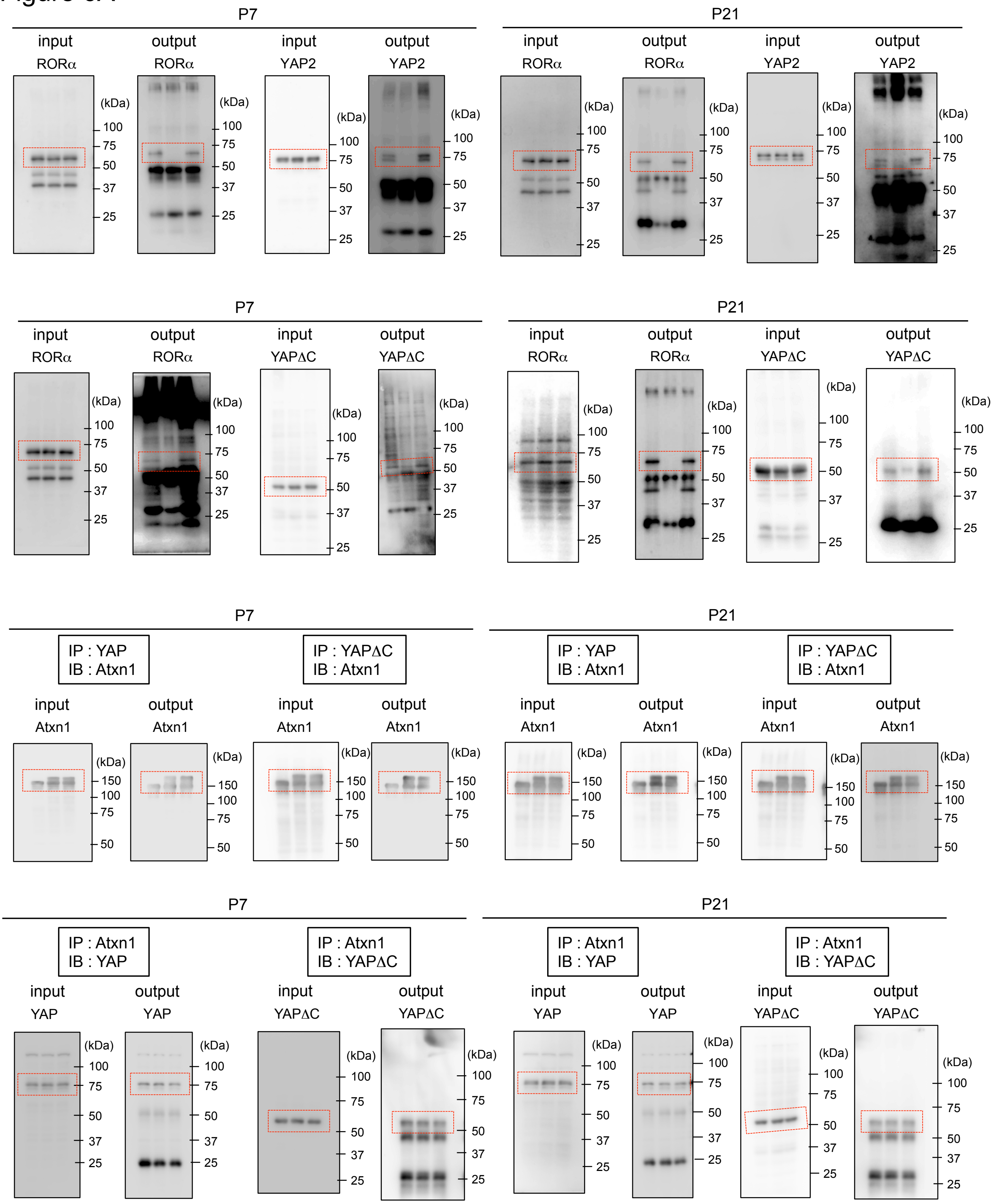
P7
P21
input
output
RORα
YAP2
YAPΔC
Atxn1
YAP
IP : YAP
IB : Atxn1
IP : YAPΔC
IP : Atxn1
IB : YAP
IB : YAPΔC
(kDa)
150
100
75
50
37
25

Figure 6B

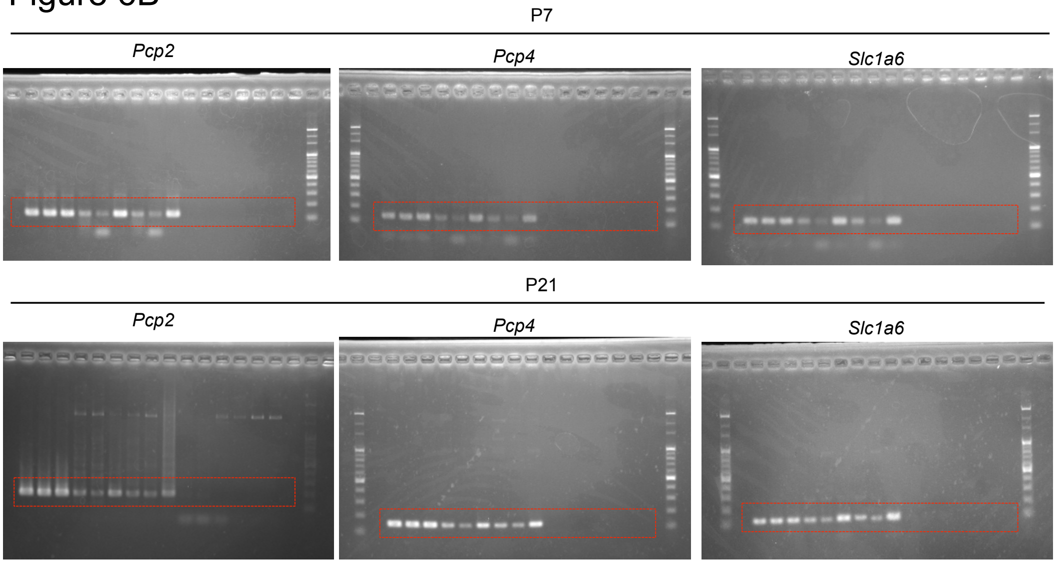

Figure 6C

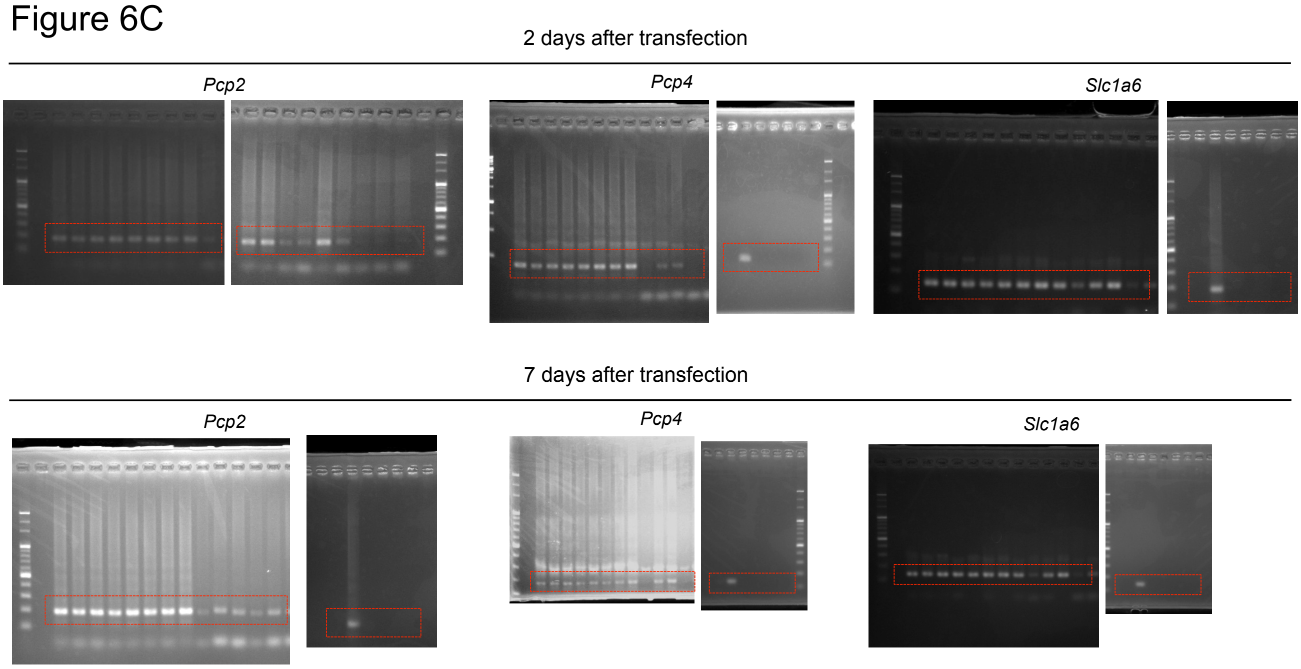

Figure 6D

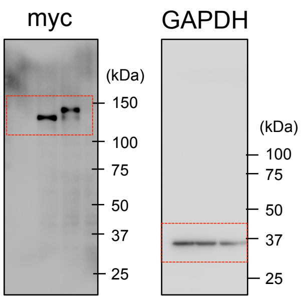

Figure 7A

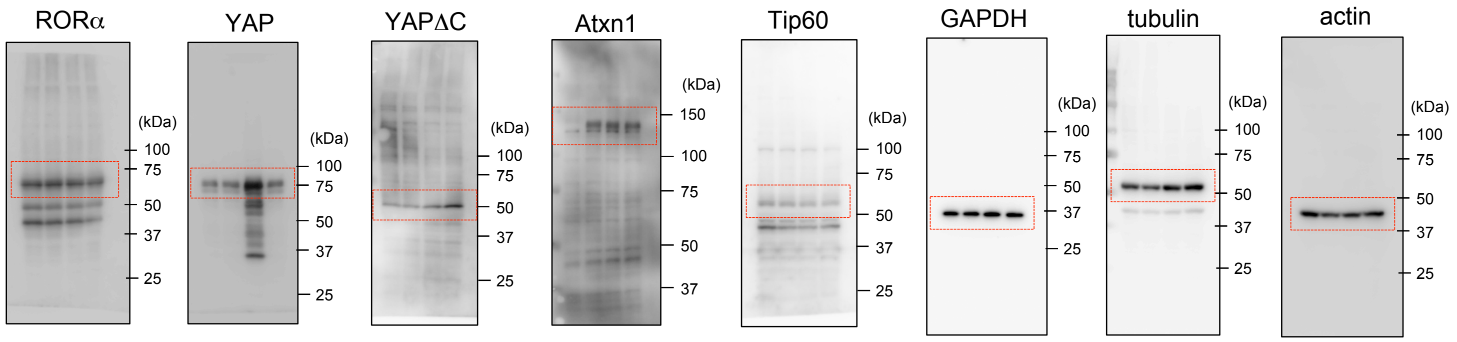

Figure 7B

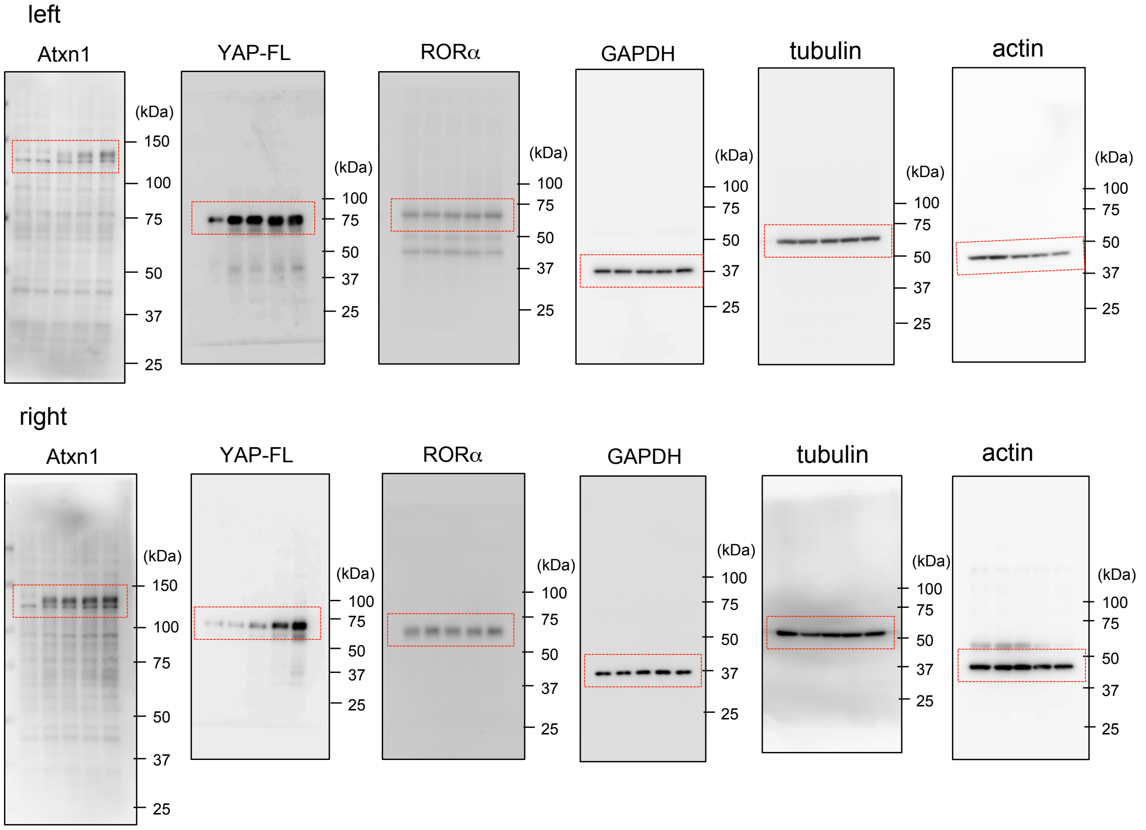

Figure 7C

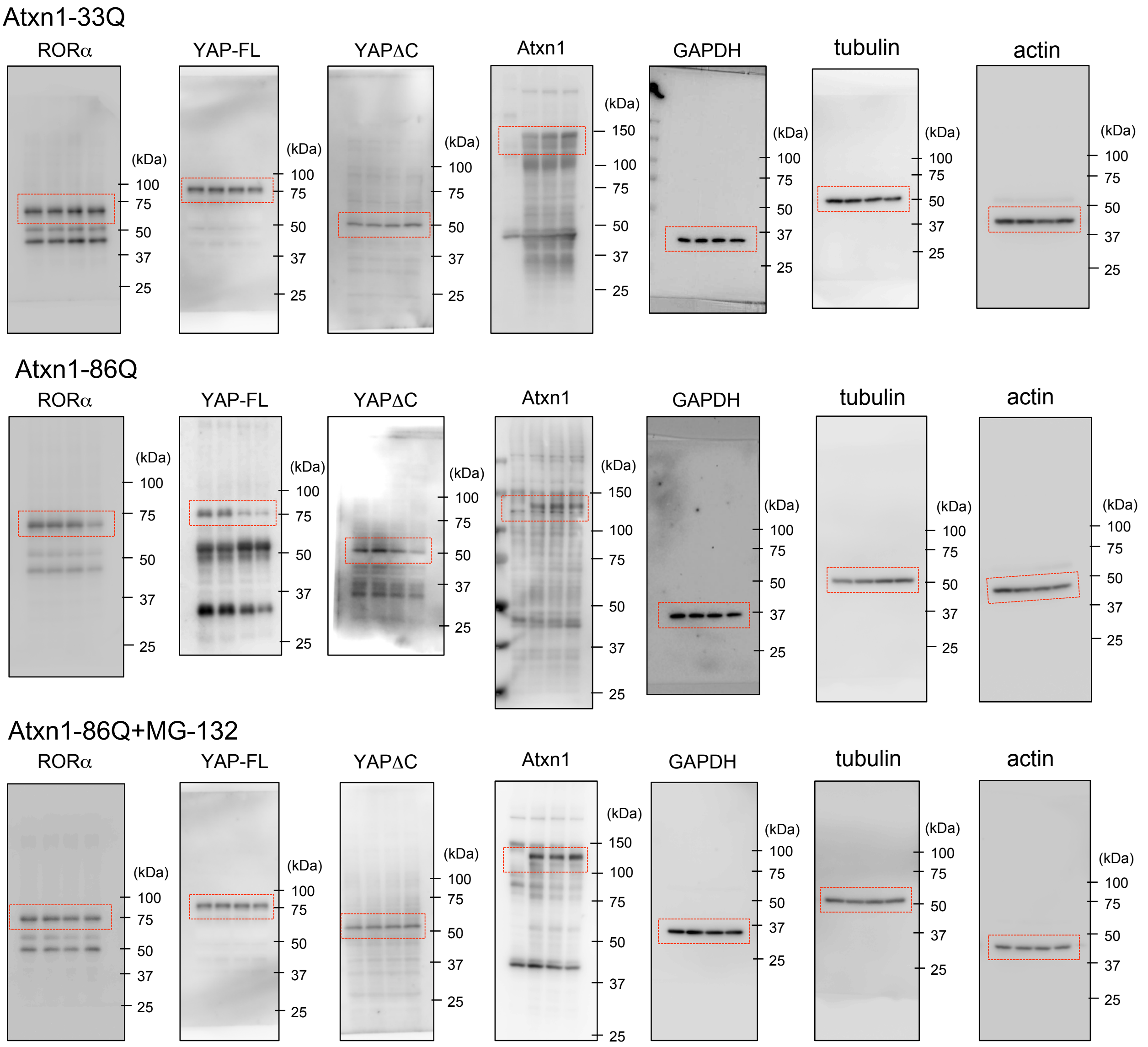
Atxn1-33Q
RORα
YAP-FL
YAPΔC
Atxn1
GAPDH
tubulin
actin
(kDa)
150
100
75
50
37
25
Atxn1-86Q
Atxn1-86Q+MG-132

Figure 8

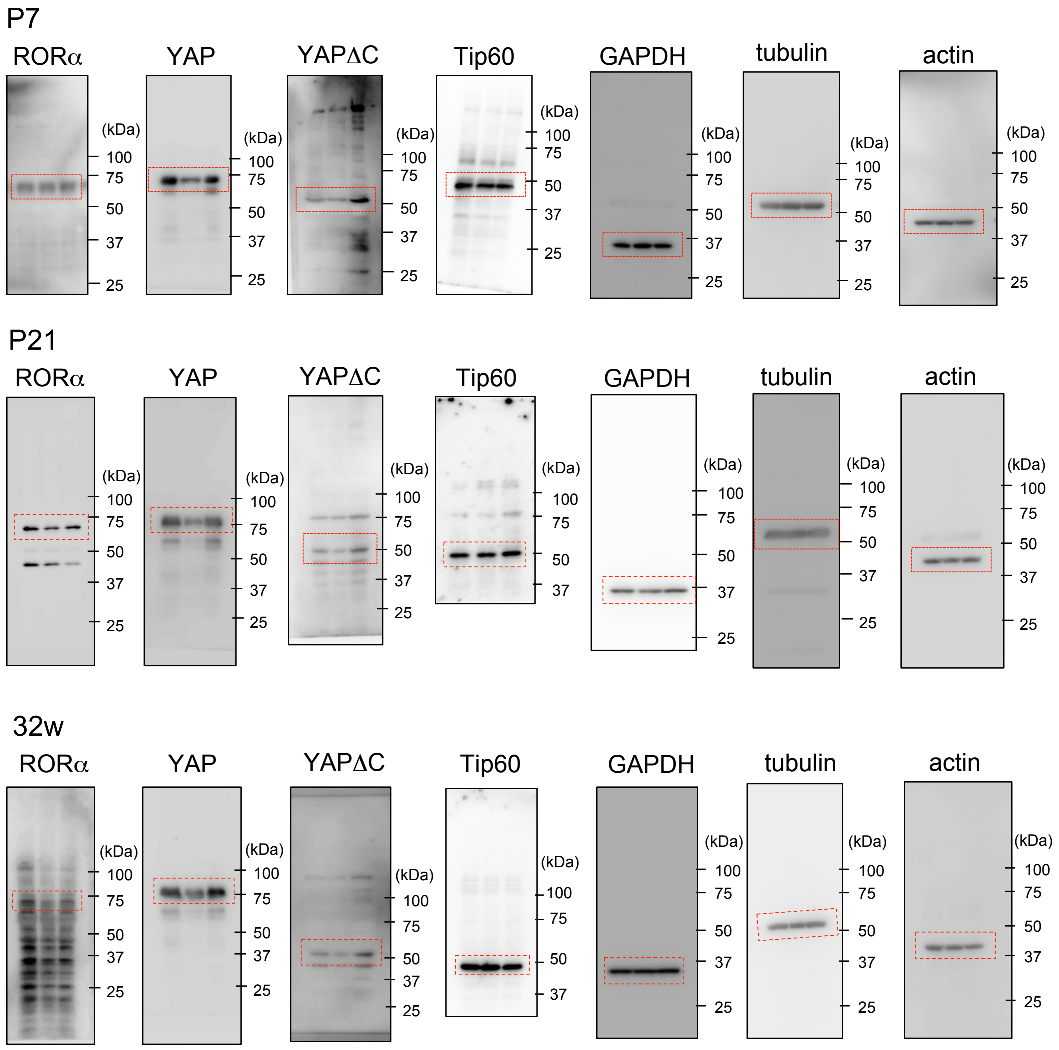
P7
RORα
YAP
YAPΔC
Tip60
GAPDH
tubulin
actin
(kDa)
100
75
50
37
25
P21
32w
